# *Pneumocystis jirovecii* pneumonia in COVID-19: an overlooked clinical entity—Response to “Pneumocystis pneumonia risk among viral acute respiratory distress syndrome related or not to COVID 19”

**DOI:** 10.1186/s13054-021-03836-7

**Published:** 2021-12-06

**Authors:** Antonio Riccardo Buonomo, Giulio Viceconte, Ivan Gentile

**Affiliations:** Department of Clinical Medicine and Surgery, University of Naples “Federico II”, Via Sergio Pansini n.5, 80130 Naples, Italy

**To the editor,**

We read with great interest the article published in Critical Care by Razazi et al. reporting no cases of *Pneumocystis jirovecii* pneumonia (PJP) among intubated patients with acute respiratory distress syndrome (ARDS) secondary to COVID-19 (C-ARDS) [1].

The authors compared these results with a historical cohort of non-COVID-19 ARDS (NC-ARDS). They showed a higher incidence of proven PJP and PCR positivity (without a diagnosis of PJP) in respiratory samples in NC-ARDS than in C-ARDS (0.05% and 13% vs 0% and 0%, respectively) [1].

However, patients in study by Razazi et al. are enrolled during the first period of pandemic, when dexamethasone was not strongly recommended and, surprisingly, they found a more profound lymphopenia in NC-ARDS. Moreover, in NC-ARDS group 82% of enrolled patients were immunocompromised compared to 13% of C-ARDS [1].

Conversely, we have published 5 cases of proven PJP in immunocompetent hosts in late phase of COVID-19 disease [2, 3]. According to EORTC/MSGERC diagnostic criteria, we observed that the use of steroids was the most frequent host factor that predispose to PJP [4].

Moreover, Razazi et al. showed that the two proven PJP diagnosis in NC-ARDS cohort had Beta-D Glucan assay (BDG) > 80, while in our experience we documented negative BDG in all the proven cases.

In the end, we think that the absence of PJP cases in C-ARDS cohort may have been influenced by the phase of COVID-19 clinical course and lower dosage of steroids administrated, while the higher prevalence of PJP diagnosis and qPCR positivity in NC-ARDS cohort should be led back to the high prevalence of immunocompromised patients enrolled.

Therefore, since either lymphopenia or steroidal treatment are strongly associated with the risk of PJP development, further studies are needed to detect any other risk factor for developing PJP in COVID-19 and to design any potential prophylactic strategies. Nevertheless, it is noteworthy that BDG assay has a high negative predictive value in HIV positive patients for PJP diagnosis, while it is less clear the real power of this test in other settings such as immunocompetent patients and COVID-19.

In conclusion, the pathogenesis of PJP in late COVID-19 and the role of BDG and of PCR in predicting development of PJP must be further investigated, and PJP should be taken into account in differential diagnosis of respiratory relapse in late COVID-19 by obtaining invasive samples (bronchoalveolar lavage), since BDG seems to have a low negative predictive value in this setting.

## Data Availability

Not applicable.
