# Supplementary material for: Pneumocystis jirovecii pneumonia in COVID-19: an overlooked clinical entity—Response to “Pneumocystis pneumonia risk among viral acute respiratory distress syndrome related or not to COVID 19”
Source: Crit Care. 2021 Dec 6;25:418. doi: 10.1186/s13054-021-03836-7 (PMC8647515; doi:10.1186/s13054-021-03836-7)
Supplement: Related texts

# Reply: Authors’ response

**Authors:** Romain Arrestier, Armand Mekontso-Dessap, Francoise Botterel, Keyvan Razazi

**To the Editor,**

We thank Buonomo and colleagues for their comment that our finding of a very low risk of *Pneumocystis* pneumonia in COVID-19 patients with acute respiratory distress syndrome (ARDS) stands in contrast to their experience on *Pneumocystis* pneumonia in COVID-19 patients.

We agree that enrolment of patients during the first wave may be a limit of our study because of a lower use of steroid therapy. To explore further this potential bias, we analyzed 239 consecutive COVID-19 patients, hospitalized in our intensive care unit during the following waves from May 26th 2020 to April 11th 2021 (*Anticovid study Clinical trials NCT04433105*). All were treated with steroids, 39 (16.3%) with tocilizumab and 29 (12.1%) were immunocompromised.

Of these, 73 had a *Pneumocystis* examination, including 131 PCR and 107 direct examinations (43 May Grunwald Giemsa stainings and 64 Grocott colorations) which were all negative. Only three patients (4%) had a positive PCR with a cycle threshold range of 35.5–37. All were classified as *Pneumocystis* colonization. Two of them did not have any pre-existing risks factors besides 10 days of steroid for COVID-19 and had a negative β-d-glucan. One patient with liver transplantation and long-term steroid therapy and calcineurin inhibitor treatment had a positive PCR with only one low titer positive β-d-glucan (178 pg/mL); he was classified as P*neumocystis* colonization because his state improved without trimethoprim-sulfamethoxazole treatment.

The five patients described by Gentile and al [2] were very different from those of our cohort. None of them had ARDS requiring invasive mechanical ventilation, and they were surprisingly all treated with steroids with a very high cumulative dose of steroid (range from 400 to 1150 mg prednisone equivalent) and a longer duration than recommended.

The authors did not specify if they received a trimethoprim-sulfamethoxazole prophylaxis. In our intensive care unit, we systematically prescribed a prophylaxis as suggested if the steroid dose is higher than 0.3 mg/kg/d equivalent prednisone for at least 3 weeks, whatever the disease [5, 6]. This could explain the difference between our observation and those from Gentile and al.

In a recent meta-analysis, a negative BDG test to ‘rule out’ PJP with 95% certainty (post-test probability of 5%) requires that the pre-test probability be low (< 20%), as in COVID-19 patients [7].

Altogether, these data confirm that even when steroid therapy was the standard of care, *Pneumocystis* pneumonia risk is very low among patients with COVID-19 related ARDS even in immunocompromised patients.
